# Diagnostic Validity of Self-Reported Hearing Loss in Elderly Taiwanese Individuals: Diagnostic Performance of a Hearing Self-Assessment Questionnaire on Audiometry

**DOI:** 10.3390/ijerph182413215

**Published:** 2021-12-15

**Authors:** Tzong-Hann Yang, Yuan-Chia Chu, Yu-Fu Chen, Meng-Yu Chen, Yen-Fu Cheng, Chuan-Song Wu, Hung-Meng Huang

**Affiliations:** 1 Department of Otorhinolaryngology, Taipei City Hospital, Taipei 100, Taiwan; tzonghannyang@gmail.com (T.-H.Y.); isendtw@gmail.com (M.-Y.C.); 2 General Education Center, University of Taipei, Taipei 10671, Taiwan; 3 Department of Medical Research, Taipei Veterans General Hospital, Taipei 112, Taiwan; yfcheng2@vghtpe.gov.tw; 4 Department of Speech-Language Pathology and Audiology, National Taipei University of Nursing and Health Sciences, Taipei 112303, Taiwan; yufuchen@ntunhs.edu.tw; 5 Information Management Office, Taipei Veterans General Hospital, Taipei 112, Taiwan; xd.yuanchia@gmail.com; 6 Department of Information Management, National Taipei University of Nursing and Health Sciences, Taipei 112, Taiwan; 7 School of Medicine, National Yang Ming Chiao Tung University, Taipei 112, Taiwan; 8 Department of Otolaryngology-Head and Neck Surgery, Taipei Veterans General Hospital, Taipei 112, Taiwan; 9 Institute of Brain Science, National Yang Ming Chiao Tung University, Taipei 112, Taiwan; 10 College of Science and Engineering, Fu Jen University, Taipei 243, Taiwan

**Keywords:** HHIE-S, age-related hearing loss, questionnaire, hearing screening, community-based study, validity, reliability

## Abstract

Key Points: Question: Can the traditional Chinese version of the hearing handicap inventory for elderly screening (HHIE-S) checklist screen for age-related hearing loss (ARHL) in elderly individuals? Findings: In this cross-sectional study of 1696 Taiwanese patients who underwent annual government-funded geriatric health checkups, the Chinese version of the HHIE-S had a sensitivity of 76.9% and a specificity of 79.8% with a cutoff score greater than 6 for identifying patients with disabled hearing loss (defined as a PTA > 40 dB). Meaning: The traditional Chinese version of the HHIE-S is an effective test to detect ARHL and can improve the feasibility of large-scale hearing screening among elderly individuals. Purpose: The traditional Chinese version of the hearing handicap inventory for elderly screening (TC-HHIE-S) was translated from English and is intended for use with people whose native language is traditional Chinese, but its effectiveness and diagnostic performance are still unclear. The purpose of this study was to evaluate the validity and reliability of the traditional Chinese version of the HHIE-S for screening for age-related hearing loss (ARHL). Methods: A total of 1696 elderly people underwent the government’s annual geriatric medical examination at community hospitals. In this cross-sectional study, we recorded average conducted pure-tone averages (PTA) (0.5 kHz, 1 kHz, 2 kHz, 4 kHz), age, sex, and HHIE-S data. Receiver operating characteristic (ROC) curve analysis was used to identify the best critical point for detecting hearing impairment, and the validity of the structure was verified by the agreement between the TC-HHIE-S and PTA results. Results: The HHIE-S scores were correlated with the better-ear pure-tone threshold averages (PTAs) at 0.5–4 kHz (correlation coefficient r = 0.45). The internal consistency of the total HHIE-S score was excellent (Cronbach’s alpha = 0.901), and the test-retest reliability was also excellent (Spearman’s correlation coefficient = 0.60, intraclass correlation coefficient = 0.75). In detecting disabled hearing loss (i.e., PTA at 0.5–4 kHz > 40 dB), the HHIE-S cutoff score of > 6 had a sensitivity of 76.9% and a specificity of 79.8%. Conclusions: The traditional Chinese version of the HHIE-S is a valid, reliable, and efficient tool for large-scale screening for ARHL.

## 1. Introduction

Hearing loss is very common among people aged 65 or older worldwide, but it is not fully understood or treated. According to statistics released by the United Nations in 2019, the proportion of the population aged more than 65 years in East Asia and Southeast Asia is expected to increase from 11% in 2019 to 24% in 2050 [1]. In Taiwan, the proportion of elderly people 65 years of age or older increased from 10.2% in 2007 to 14.6% in 2017 [2]. The proportion of the urban population made up of older people over 65 years of age increased from 2.33% in 1968 to 17.2% at the end of 2018 [3]. Therefore, in Taiwan and other countries, the increasing number of age-related hearing loss (ARHL) cases among elderly people is becoming an increasingly important public health problem.

There is evidence that patients with hearing loss have more tinnitus and balance problems and a worse long-term quality of life [4,5]. These findings raise serious concerns about other negative health consequences, including [6] walking difficulties [7], driving ability [8], social isolation [9], cognition [9,10], dementia [11,12], functional decline [13,14], falls [15], increasing disability-adjusted life years (DALYs) [16], and mortality [13]. Detecting and even treating ARHL are therefore key components of healthy aging. Prior studies have used subjective measures, such as a manual audiometry, test to detect ARHL [17,18]. However, a cost-effective approach for large-scale hearing screenings is still lacking, which is particularly concerning given that the number of older individuals is increasing, and there is a shortage of hearing and geriatric healthcare professionals [19,20]. Some studies have utilized telemedicine methods, such as telephone-based [21], computer-based [22,23], internet-based, or smartphone-based applications [24,25,26], to screen for hearing impairment in elderly individuals. Moreover, questionnaires can provide inexpensive, efficient, and truly self-perceived measurements of hearing loss.

Hearing loss is usually defined by the difference in threshold between the two ears. There are many recommended standards for comparative hearing standardization of the test procedures [27], such as HST [28,29,30], AAP [31], and ASHA [32]. For example, the American Association for Speech Language Hearing (ASHA) and the American Academy of Audiology recommended that 20 dB screening be performed at frequencies of 1000 Hz, 2000 Hz, and 4000 Hz [32]. In 2003, the American Academy of Pediatrics (AAP) also recommended screening at 20 dB at frequencies of 500 Hz, 1000 Hz, 2000 Hz, and 4000 Hz [31]. In HST [28,29,30], different hearing levels are also divided into grade 1 (PTA ≤ 25 dB HL), grade 2 (PTA 26–45 dB HL), grade 3 (PTA 46–75 dB HL), grade 4 (PTA 76–90 dB HL), and grade 5 (PTA > 90 dB HL).

The hearing handicap inventory for elderly screening (HHIE-S) is an abbreviated version of the elderly hearing impairment inventory (HHIE). It consists of 10 questions that can help clinicians assess the emotional and social aspects of hearing loss. The HHIE-S score has been verified with respect to a hearing test for hearing loss, and the results show that it has sufficient sensitivity and specificity in identifying individuals with hearing loss [33,34]. Yang et al. translated the traditional Chinese version of the HHIE-S (TC-HHIE-S) from English to assess the disability and hearing loss of elderly individuals whose native language is traditional Chinese [35]. However, its diagnostic validity and reliability are still unknown.

## 2. Methods

### 2.1. Study Population

The subjects included in this cross-sectional study were individuals residing in the Zhongzheng and Wanhua districts of Taipei City, which consist of 348,390 residents, 18.5% of whom are older adults aged more than 65 years. Taipei is the most populated city in Taiwan and is comprised of 12 districts with a total population of approximately 2.7 million [3]. The Heping branch of the Taipei City Hospital is a community hospital that typically provides health care for local residents. The subjects included in this study were enrolled from an annual government-funded geriatric health check-up program that is free for all Taipei citizens aged more than or equal to 65 years old. The eligible elderly individuals were informed by the city government about this program, and the health check-up services were typically arranged at a community hospital based on their residential area. The cohort of this study comprised participants who underwent a health check-up at the Heping branch of the Taipei City Hospital, which is a community hospital that provides healthcare services for people living in Zhongzheng and Wanhua districts of Taipei. A total of 1696 adults, 47% (797/1696) male and 53% (899/1696) female, were enrolled (*p* > 0.05). This study was approved by the Joint Institutional Review Board of Taipei City Hospital (TCHIRB-10811011-E).

### 2.2. Setting and Procedures

The data used in this community-based study were collected from the Health Promotion Center of the Heping branch of the Taipei City Hospital from 2016 to 2018. The sample size was estimated considering the type I error rate and type II errors using 0.05 (two-tailed test) and 0.2 (power = 80%), respectively. The required sample size was 886 participants, as calculated by two independent *t*-tests, which corresponds to the small effect size of d = 0.2, and the allocation ratio was 1:2. Therefore, the sample size used in this study had enough power to detect differences between the groups. We recruited 1696 people aged 65 or older who visited an otolaryngology outpatient clinic. Investigators explained the research objectives and process, and written informed consent was obtained from all the patients who were enrolled. Instructions regarding the screening procedures and operations were provided by the trained examiners prior to each hearing screening test.

The participants met the following requirements: (1) self-suspected hearing loss and (2) able to understand and communicate in Chinese or Taiwanese. Patients were excluded if they had any neurological diseases that might interfere with their decision making. All of the subjects included in the study were randomly recruited. Informed consent was obtained from the participants, and then, demographic information, such as age and sex, was collected via a questionnaire. The TC-HHIE-S was then administered by a licensed interviewer, an audiologist, and a trained undergraduate student in audiology supervised by the audiologist. The order of the questions of the TC-HHIE-S is the same as that of the English version. First, each participant was given general instructions for the TC-HHIE-S by the interviewer. The items were then administered via a face-to-face interview. A score of 0 was assigned for “no” responses, a score of 2 was assigned for “sometimes” responses, and a score of 4 was assigned for “yes” responses. The total score was the sum of all the scores for the responses. The air conduction pure-tone average at hearing thresholds of 0.5, 1, 2, and 4 kHz was then assessed with an MA30 audiometer (Maico, Germany) using pure-tone stimuli in a sound-treated booth with ambient noise <30 dBA located in a quiet room. The thresholds were measured with standard TDH-39 supra-aural earphones and expressed in dB HL; the thresholds were considered the intensity levels at which the tones were heard in 50% of the trials using the descending technique. If a subject reported asymmetric hearing, the better ear was tested first. Otherwise, the right ear was tested first. Narrow band masking was used as appropriate. An ordinary patient-response button was used by nearly all the subjects. The pure-tone average (PTA) was calculated by averaging the measurements of these four frequencies’ AC thresholds. The PTA of the better-hearing ear was used for the final analysis. Calibration was performed according to the reference equivalent sound pressure levels specified by ISO 389-1 and 389-3. All equipment was calibrated annually.

## 3. Statistical Analysis

IBM ©SPSS © statistics version 25 (IBM Corp., Armonk, NY, USA), R 3.6.1 (R Foundation for Statistical Computing, Vienna, Austria), and Microsoft Excel 2013 (Microsoft Corp., Redmond, WA, USA) were used for all the statistical analyses in this study. For the descriptive data, we calculated the mean and standard deviation. For the categorical data, we used chi-square tests. For continuous data, we used the Mann–Whitney U test instead of the independent *t*-test when the data were not normally distributed.

Construct validity was verified by the agreement between the TC-HHIE-S and PTA results, as determined using Spearman’s correlations (Rho); the PTA was used as the gold standard of hearing sensitivity. The reliability of the TC-HHIE-S was estimated in terms of internal consistency with Cronbach’s alpha, with a value of 0.7 being the minimally acceptable level. The test-retest consistency was evaluated with the data from a total of 188 subjects who repeated the audiometry tests and TC-HHIE-S with PTA changes of less than 10 dB HL during the study period. Spearman’s correlation coefficients and intraclass correlation coefficients (ICCs) with corresponding 95% confidence intervals were calculated. Usually, ICCs greater than 0.7 are considered to indicate good repeatability. A receiver operating characteristic (ROC) curve was utilized to find the optimal cutoff point for detecting hearing impairment. The sensitivity and specificity were also calculated.

## 4. Results

This study included 1696 adults who visited an emergency department or an otolaryngology clinic. The mean age of the study cohort was 75.0 ± 21.0 years, and 53% (899/1696) were females. The responses to the TC-HHIE-S are shown in Table 1. The majority of the subjects responded to each item with “no” (47.6–87.6%), followed by “sometimes” (5.0–23.7%) and “yes” (2.3–17.5%).

Table 2 demonstrates the demographic data stratified by the better-ear pure-tone average. There were 1001 subjects with a better-ear PTA ≤ 25 dB HL and 695 subjects with a better-ear PTA > 25 dB HL. Those with a better-ear PTA > 25 dB HL tended to be older, were significantly more likely to be male, and had a significantly higher average TC-HHIE-S score.

For construct validity, the Spearman’s correlation coefficient was approximately 0.45 between the total score of the TC-HHIE-S and the audiometry results, which means that they were moderately correlated (Table 3). However, when the results for each item of the TC-HHIE-S were compared with the audiometry results, only weak correlations were found. On the other hand, the internal consistency of the TC-HHIE-S was quite strong, with a Cronbach’s alpha of 0.901.

Table 4 shows the test-retest reliability of the TC-HHIE-S. For the total score, we found that it had good repeatability (ICCs > 0.7).

**Figure 1:**
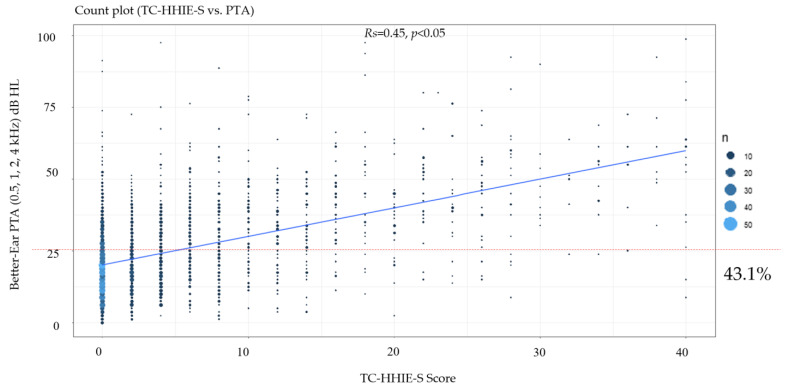
Relationships of TC-HHIE-S scores with better-ear PTAs (0.5, 1, 2, 4 kHz).

Figure 1 illustrates the relationship between the TC-HHIE-S and the audiometry results. The Spearman’s correlation coefficient was 0.45, which means that the results for the two methods were moderately correlated. We also found that 43.1% of the subjects with normal hearing felt that their hearing was impaired according to the TC-HHIE-S results.

**Figure 2:**
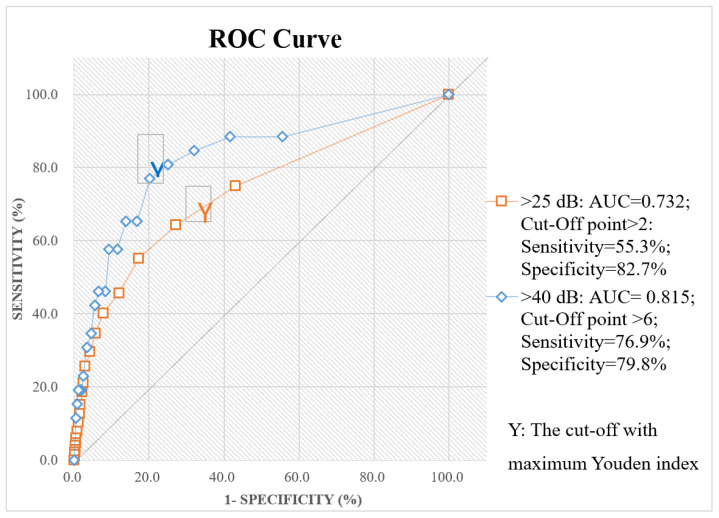
Receiver operating characteristic curves for the TC-HHIE-S scores and different definitions of hearing impairment.

The ROC curves for the TC-HHIE-S scores and the different definitions of hearing impairment are shown in Figure 2. In the figure, the red line represents the assessment of ears with better hearing with PTAs worse than 25 dB HL. The area under curve (AUC) was 0.732. For the TC-HHIE-S, when the cutoff point was greater than 2, the sensitivity was 55.3%, and the specificity was 82.7%. These results are unsatisfactory. However, as shown by the blue line in Figure 2, the TC-HHIE-S is a suitable tool for identifying patients with disabling hearing loss, i.e., those in whom the better-ear pure-tone average threshold is greater than 40 dB HL. Considering the blue line, the AUC was 0.815. When the cutoff point was greater than 6, the TC-HHIE-S showed a sensitivity of 76.9% and a specificity of 79.8%.

## 5. Discussion

To the best of our knowledge, this is the first community-based study to evaluate the effectiveness of TC-HHIE-S in the diagnosis of age-related hearing loss among elderly individuals in Taiwanese Mandarin. We aimed to assess the utility of the TC-HHIE-S as a screening tool for identifying Mandarin-speaking elderly individuals with disabled hearing loss/hearing impairment who may need otologic or audiologic referrals because aural rehabilitations are warranted. This study investigated the relationship between audiometric and self-assessed hearing measurements determined by the 10-item Likert-type TC-HHIE-S questionnaire.

In this study, we measured the construct validity of the TC-HHIE-S and found a Spearman’s correlation coefficient of 0.45 (Table 3, Figure 1). This result indicated a weak-to-moderate correlation between actual hearing loss and self-perceived hearing impairment. Although Spearman’s correlation coefficient was not high, it was only slightly lower than that reported in a prior study conducted in Taipei and better than that reported in a Swedish study (Table 5). The inconsistency among these studies regarding the correlation of the audiometric and the self-reported hearing impairment measurements was most likely caused by differences in the study cohorts (such as discrepancies in age composition) and differences in the languages used for evaluating self-perceived hearing impairment. The cultures of, ethnicities of, and sampling methods used for the different populations may also have affected the results. The weak-to-moderate correlation between the pure-tone audiometry and self-reported handicap results in the present study suggests that the self-reported assessment could be useful for hearing screenings in large populations.

The internal consistency of the total score of the TC-HHIE-S was excellent in this study, with a Cronbach’s alpha of 0.901 [42], despite the internal consistency of items 1, 2, 4, and 10 being questionable; the internal consistency of items 5 and 9 being poor; and the internal consistency of items 6 and 7 being unacceptable. The internal consistency of item 8 was acceptable, and the internal consistency of item 3 was good (Table 3). The internal consistency of the TC-HHIE-S total score in this study also had good agreement with the values reported in previous studies using different languages (Table 5).

We found that the Spearman’s correlation coefficient (Spearman’s r) was 0.60, and the intraclass correlation coefficient (ICC) was 0.75 (95% CI = 0.67–0.81) when the TC-HHIE-S scores for the first and second administrations were compared to estimate test-retest reliability (Table 4). In comparison to prior studies, our study reported the lowest ICC value because the 188 subjects who were tested at a certain time and retested in the following year with a different interviewer had better-ear PTA differences of less than 10 dB (Table 5). The long interval between the test and retest favorably eliminated the memory effect of the administered questionnaire. However, it was impossible to prevent the effects of maturation, such as the possibility of higher hearing threshold decrements, in the following year. Nevertheless, the ICC in our study can still be interpreted as good [43] or excellent [44]. Moreover, the longer time interval between the two questionnaire administration times and the tests being conducted by different interviewers might account for the lower ICC in this study (Table 6).

Screening for any disease typically leads to an increase in the likelihood that individuals with the disease will be identified (sensitivity), and those without the disease (specificity) will be excluded. In practice, however, not all subjects will be identified by screening (i.e., false negatives), and some individuals without the disease may be wrongly classified as having it (i.e., false positives). Therefore, there is an intrinsic and inevitable trade-off between sensitivity and specificity [45]. According to the results of this study, a cut-off value of >6 corresponded to a sensitivity of 76.9% and a specificity of 79.8% for identifying disabled hearing loss (PTA > 40 dB HL). An increase in the cutoff point to >8 led to a sensitivity of 80.8% and a specificity of 75.0%. An additional increase in the cutoff point to >10 led to a sensitivity of 88.5% and a specificity of 58.3% (Figure 2). PTA > 40 dB HL had the highest sensitivity (76.9%) in hearing screenings, while PTA > 25 dB HL showed the highest specificity (82.7%) (Figure 2).

Choosing the optimal cut-off point for large-scale hearing screenings warrants additional studies. Table 5 shows the different cut-off points of the HHIE-S used in previous studies for diagnosing disabled hearing loss. The cut-off points varied from 6 to 18 and yielded various sensitivity and specificity results. The sensitivity and sensitivity results of our study are comparable to those of a Chinese study conducted in 2017. Although some studies revealed higher sensitivity and specificity values, this finding may be due to differences in the study protocol, such as the cut-off value used for diagnosing hearing impairment, the language of the assessment, the numbers of subjects, the test setting, the interview method, the hearing loss prevalence among the study cohort, and the race/ethnicity and cultural experiences of the study participants.

The TC-HHIE-S is a cost-effective and easy test to administer in various settings, including senior centers, nursing homes, and primary care practices, where standard audiometry is often not available. This study suggests that the TC-HHIE-S can be important for public health applications because of its ability to detect moderate and severe hearing loss in a large population. To further improve the feasibility and efficiency of the HHIE-S for large-scale screening, future studies that administer the TC-HHIE-S via internet or smartphone-based approaches are needed.

The TC-HHIE-S proposed in this study has been shown to be suitable for hearing screening in primary care practices (PCPs) or other busy clinical settings, such as urgent care and emergency departments for individual care. We believe that this TC-HHIE-S can be used as a point-of-care test for PCP-level hearing screening because it is economical and efficient and requires minimal training of case managers in health systems.

There are several strengths of our study. The cohort included in this study is a representative sample of noninstitutionalized older adults in Taiwan because they were randomly selected among individuals eligible for the global annual government-funded geriatric health check-up program. The cost of audiometric testing was covered by the research grant that supported this study, and thus, no out-of-pocket expenses were paid by the study participants. As a result, the prevalence of hearing loss was not confounded by socioeconomic factors. The use of standard audiometry equipment in a sound-treated room also reduced the potential measurement errors caused by ambient noise.

Nonetheless, the study also has several limitations that need to be addressed. The participants of this study voluntarily agreed to join the annual health check-up program. Selection bias may be present, and the results of our study may not be generalizable to less healthy or hospitalized populations.

## 6. Conclusions

In this study, the diagnostic reliability and validity of the TC-HHIE-S for identifying individuals with age-related hearing loss were proven to be acceptable. We believe that the TC-HHIE-S is an effective and cost-effective tool for large-scale hearing screenings among older adults. Additional clinic-epidemiological studies of the administration of the TC-HHIE-S via webpages or smartphone apps are thus imperative.

## Figures and Tables

**Table 1:** Subject’ Responses to the Traditional Chinese HHIE-S (TC-HHIE-S).

	Patient Responses (Number, %)			
Item	No	Sometimes	Yes	No Response
1. Do hearing problems embarrass you when you meet new people?	1318 (77.7)	251 (14.8)	108 (6.5)	19 (1.1)
2. Does a hearing problem make you feel frustrated when you talk to members of your family?	1328 (78.3)	248 (14.6)	101 (6.0)	19 (1.1)
3. Do you find it difficult hearing when someone speaks in a whisper?	977 (47.6)	402 (23.7)	297 (17.5)	20 (1.2)
4. Do you feel that you have a disability because of a hearing problem?	1292 (76.2)	224 (13.2)	161 (9.5)	19 (1.1)
5. Does a hearing problem cause you difficulty when visiting friends and relatives or neighbors?	1431 (84.4)	159 (9.4)	87 (5.1)	19 (1.1)
6. Does a hearing problem reduce your attendance at religious ceremonies more than you would like?	1519 (89.6)	84 (5.0)	71 (4.2)	22 (1.3)
7. Does a hearing problem cause disputes between you and a family member?	1503 (88.6)	135 (8.0)	39 (2.3)	19 (1.1)
8. Does a hearing problem cause difficulty when you are listening to the radio or television?	1161 (68.5)	331 (19.5)	183 (10.8)	21 (1.2)
9. Do you feel that any difficulty in hearing limits or hinders your personal or social life?	1488 (87.6)	125 (7.4)	66 (3.9)	19 (1.1)
10. Does a hearing problem cause you difficulty when you are in a restaurant with relatives or friends?	1335 (78.7)	220 (13.0)	120 (7.1)	21 (1.2)

**Table 2:** Demographic Data of the Patients with a Better-Ear PTA (0.5, 1, 2, 4 kHz) > 25 dB HL and ≤ 25 dB HL).

Variable	≤25 dB HL (*n* = 1001)	>25 dB HL (*n* = 695)	*p*-Value
Age (years)	71.8 ± 5.8	79.5 ± 31.5	<0.001 *
Sex, *n* (%)			<0.001 *
Male	391 (39.1%)	406 (58.4%)	
Female	610 (60.1%)	289 (41.6%)	
TC-HHIE-S score (mean ± SD)	2.65 ± 5.0	9.55 ± 10.1	<0.001 *

The values are the mean ± SD or *n* (%), and the Mann–Whitney U test or chi-square test was used where appropriate. * statistically significant.

**Table 3:** Construct Validity and Reliability of the TC-HHIE-S.

Construct Validity			Reliability	
Spearman Correlations between the TC-HHIE-S Item and Audiometry Results			The Internal Consistency of the TC-HHIE-S	
Item	r	*p*-Value	Corrected Item: Total Correlation	Cronbach’ s Alpha (If Item Deleted)
1	0.35	0.00	0.63	0.89
2	0.35	0.00	0.67	0.89
3	0.42	0.00	0.81	0.9
4	0.37	0.00	0.67	0.89
5	0.37	0.00	0.59	0.89
6	0.32	0.00	0.48	0.89
7	0.22	0.00	0.43	0.9
8	0.37	0.00	0.74	0.89
9	0.32	0.00	0.53	0.89
10	0.30	0.00	0.64	0.89
Total score	0.45	0.00	Cronbach’s alpha	0.9

**Table 4:** Test-retest Reliability Descriptive Statistics and Repeatability Measures of the TC-HHIE-S.

	Mean Score of the TC-HHIE-S			
Item	First Administration	Second Administration	Spearman’s r	ICC * (95% CI **)
1: score	0.70 ± 1.21	0.50 ± 1.09	0.36	0.59 (0.46–0.70)
2: score	0.67 ± 1.26	0.57 ± 1.16	0.25	0.47 (0.29–0.60)
3: score	1.06 ± 1.50	1.34 ± 1.69	0.39	0.56 (0.42–0.67)
4: score	0.45 ± 1.09	0.99 ± 1.52	0.31	0.52 (0.34–0.65)
5: score	0.44 ± 1.10	0.51 ± 1.19	0.44	0.64 (0.52–0.73)
6: score	0.24 ± 0.86	0.22 ± 0.81	0.41	0.62 (0.49–0.71)
7: score	0.33 ± 0.90	0.20 ± 0.70	0.27	0.41 (0.21–0.56)
8: score	0.78 ± 1.35	0.99 ± 1.44	0.31	0.53 (0.37–0.65)
9: score	0.39 ± 1.06	0.35 ± 1.01	0.38	0.57 (0.43–0.68)
10: score	0.56 ± 1.19	0.78 ± 1.33	0.27	0.40 (0.20–0.55)
Total score	5.64 ± 8.71	6.45 ± 8.87	0.60	0.75 (0.67–0.81)

* ICCs: Intraclass correlation coefficients. ** CI: Confidence interval. ICCs > 0.70 were considered to indicate good repeatability.

**Table 5:** HHIE-S Cut-off Point, Sensitivity, and Specificity of Various Studies.

Study	Language/Place	N	Setting	Interview Method	Hearing Loss Criteria and Prevalence (%)		Cutoff Point	AUC	Rs	Sen (%)	Spec (%)
Lichtenstein, 1988 [36]	English/Nashville, Tenn	304	4 university- and 2 community-based internist practices	-	1. Ventry criteria	30	>8	-	-	72	77
					2. SFPTA	38				66	79
					3. HFPTA	58				53	84
					4. SRT	29				62	72
					5. NU 6	24				63	72
Nondahl, 1998 [37]	English/Beaver Dam Township, Wis	3556	Population-based	-	Worse-ear PTA (0.5–4 kHz) > 25 dB	45.0	>8	-	-	34	95
Sindhusake, 2001 [34]	English/Sydney, Australia	2015	Population-based study	-	Better-ear PTA (0.5–4 kHz) > 40 dB	13.4	>6	0.86	-	85.5	31.0
Salonen, 2011 [38]	Finnish/Turku	164	Radom sample	Phone	Better-ear PTA (0.5–4 kHz) > 40 dB	15.2	>18	0.94	-	96.0	81.3
Deepthi, 2012	India/local language	175	House-to-house survey	-	Better-ear PTA (0.5–4 kHz) > 40 dB	25.1	>10	0.75	-	63	90.7
Diao, 2014 [39]	Simplified Chinese/Beijing	727	Retirement facilities	-	Better-ear PTA (0.5–4 kHz) > 40 dB	-	>6	-	0.48	100	84.5
Weinstein, 2015 [40]	Arabic/Cairo	100	Hospital	Written or face-to-face	Better-ear PTA (0.5–4 kHz) > 40 dB	16.0	>12	0.953	0.68	83	87
Wang, 2017 [41]	Simplified Chinese/Jilin	650	Annual health exam	Written	Better-ear PTA (0.5–4 kHz) > 40 dB	84.5	>8	-	-	84.5	58.3
This study, 2019	Traditional Chinese/Taipei	1696	Annual health exam	Face-to-face	Better-ear PTA (0.5–4 kHz) > 40 dB	15.2	>6	0.815	0.450	76.9	79.8

**Table 6:** Construct Validity and Reliability of HHIE-S in Various Studies.

Study	Language	Age	Construct Validity		Reliability		
			PTA vs. HHIE-S		Internal Consistency	Test-Retest	
			N	Rs	Cronbach’s α	*n*	ICC *
Weinstein, 1983	English		100	0.61	-	-	-
Weinstein, 1986	English		?	-	0.87	-	0.84
Chang, 2009	Traditional Chinese	>65	1220	0.52	-	-	-
Tomioka, 2013	Japanese		197	0.9	0.91	197	0.85
Diao, 2014	Simplified Chinese	60–86	727	0.75	-	-	-
Weinstein, 2015	Arabic		100	0.69	0.9	100	0.98
Oberg, 2016	Swedish		69	0.36	0.77	-	-
This study, 2019	Traditional Chinese	>65	1696	0.45	0.9	188	0.75

* ICCs: Intraclass correlation coefficients.

## Data Availability

Data not available due to ethical restrictions.
